# Role of specialized pro-resolving lipid mediators in pulmonary inflammation diseases: mechanisms and development

**DOI:** 10.1186/s12931-021-01792-y

**Published:** 2021-07-14

**Authors:** Ailin Yang, Yanjun Wu, Ganggang Yu, Haoyan Wang

**Affiliations:** Department of Respiratory Medicine, Beijing Friendship Hospital, Capital Medical University, No. 95 Yong An Road, Xicheng, , Beijing, 100050 China

**Keywords:** Inflammation resolution, Lung diseases, Lung inflammation, Pro-resolving lipid mediators, Mechanism

## Abstract

Inflammation is an essential mechanism of various diseases. The development and resolution of inflammation are complex immune-modulation processes which induce the involvement of various types of immune cells. Specialized pro-resolving lipid mediators (SPMs) have been demonstrated to be signaling molecules in inflammation. SPMs are involved in the pathophysiology of different diseases, especially respiratory diseases, including asthma, pneumonia, and chronic obstructive pulmonary disease. All of these diseases are related to the inflammatory response and its persistence. Therefore, a deeper understanding of the mechanisms and development of inflammation in respiratory disease, and the roles of the SPM family in the resolution process, might be useful in the quest for novel therapies and preventive measures for pulmonary diseases.

## Introduction

Inflammation is a major defense mechanism of the human body. If the body is subjected to an injury, infection, or other similar stimuli, the innate immune system becomes activated within seconds-to-minutes. Innate immunity is useful in promoting inflammation and can aid in determining the level of pathogenic invasion or stimuli. Although inflammatory reactions evolve as an adaptive reaction that could restore homeostasis, a successful inflammatory response is complete only after resolution and restoration phases [1, 2]. If inflammation persists or becomes uncontrolled, it could elicit an overprotective response by the body. Thus, inflammatory factors can mediate an auto-immune reaction to attack healthy cells and tissues in the body. This action can cause rheumatoid arthritis, inflammatory bowel disease, psoriasis, asthma, and vasculitis [3, 4]. Recently, studies have shown that chronic, persistent inflammation might be the cause of cancer, neurodegenerative diseases [5] (e.g., Alzheimer’s disease, Parkinson disease) and even certain types of mental illness [6].

Acute inflammation is induced by the innate immune system, which has an essential protective role, prevents some infections, and promotes healing in injured tissues. Briefly, if the body is stimulated by inflammation, infection, or other pathogenic factors, an acute immune response is accelerated. Inflammatory cells, such as polymorphonuclear neutrophils (PMNs), macrophages, and dendritic cells (DCs), are recruited by cytokines and chemokines, which accelerate the inflammatory response and reduce further damage to tissues [7].

After the body initially launches an immune response, acute inflammation begins to subside. Phagocytic cells (e.g., macrophages and neutrophils) are mobilized to eliminate apoptotic and necrotic cells and thereby promote the repair/recovery of tissue and maintain tissue homeostasis by generating pro-inflammatory mediators. In health, this process occurs actively in a specific self-limiting manner. However, if the acute inflammatory response is not controlled or completed within a specific timeframe, inflammation may persist, or the body may initiate an exaggerated inflammatory response, which may lead to inflammation recurrence. Such situations may induce chronic inflammation, disease, or even death [7].

Studies [8, 9] have determined that a series of endogenous lipid mediators produced during the resolution of acute inflammation can bind to specific receptors, inhibit neutrophil infiltration, regulate the formation of cytokines and chemokines, and thereby promote extensive phagocytosis. Endogenous lipid mediators, which can eliminate apoptotic cells and necrotic cells and promote tissue repair, can be referred to collectively as “specialized pro-resolving lipid mediators” (SPMs). They include resolvin, protectin, maresins, and lipoxins (LXs), which are converted from omega-6 polyunsaturated fatty acids [10–12]. Animal experiments have demonstrated that SPMs can be involved in the regulation of inflammatory diseases such as arthritis, peritonitis and asthma, as well as ischemia–reperfusion injury and inflammatory pain [10, 13, 14].

The respiratory system connects the internal and external environment, and can be affected readily by stimuli such as smoke (and other forms of air pollution) and pathogens. The respiratory system also serves as a natural defense system. As various stimuli enter and accumulate in the body's internal environment, they can provoke an inflammatory response in cells and tissues and may even cause a persistent reaction.

A decline in the ability to resolve inflammation fully will cause dysfunction in epithelial cells and alveolar macrophages. This dysfunction constitutes a major mechanism for the persistence of inflammation, as well as further injury to tissues and disruption of normal structure in the pulmonary parenchyma. This dysfunction may even cause asthma, chronic obstructive pulmonary disease (COPD), or pulmonary fibrosis [3, 15]. Therefore, it is especially important to recognize the relationship between inflammation resolution and the pathogenesis of respiratory diseases.

Recently, studies focusing on the decline in levels of SPMs during the early inflammatory response have demonstrated that these mediators could act as novel targets for the treatment of pulmonary diseases [16–18]. Therefore, understanding the mechanism of and resolution of inflammation will aid understanding of the role of SPMs in the early phase of therapy.

In this review, we discuss and update recent research on SPMs in pulmonary inflammation to provide new concepts for the treatment and prognosis of inflammatory disease. In addition, we discuss the SPMs family and their role in the regulation of respiratory diseases.

## Inflammation and resolution: mechanism

### Inflammatory response

The inflammatory response is a series of immune responses to inflammatory stimuli. A series of complex inflammatory-response mechanisms can protect the human body from the potential harm caused by infection or injury.

If pathogenic bacteria invade and cause damage to cells and tissues, pattern recognition receptors such as toll-like receptors or nod-like receptor expressed by immune cells can recognize pathogenic organisms (pathogen-associated molecular patterns) and damage signals (damage-associated molecular patterns) rapidly and transmit these “danger signals” to the interface of cells and tissues [19]. These signals promote the activation of innate immune cells (neutrophils, macrophages, eosinophils, mast cells, natural killer cells, γδ-T cells, innate lymphoid cells, and DCs), to promote them produce some pro-inflammatory cytokines and chemokines [20, 21]. These chemokines activate the chemotaxis process to recruit leukocytes and macrophages into the site of inflammation center to provide effector functions [22].

Simultaneously, under the influence of vasoactive amines (e.g., histamine) and eicosanoids (e.g., prostaglandins, 5-hydroxyeicosatetraenoic acid), granulocytes and macrophages can up-regulate the expression of complement (C3) and immunoglobulin (Ig) receptors to enhance phagocytosis and cell-killing ability [23]. In addition, inflammation and injury effectively enhancing the immune response and vascular permeability, antibodies and some soluble substances, such as SPMs and its analogs, produced by adaptive immune cells (T and B lymphocytes) flow into the center of the inflammatory response with blood plasma to form an acquired immune response. Furthermore, some structural cells in the body, such as airway and alveolar epithelial cells, endothelial cells, and fibroblasts, are also involved in the immune response during the inflammatory response [24]. These structural cells are mainly involved in the production of pro-resolving mediators and adaptive immune responses.

### Inflammation resolution

In health, the body has a complete regulatory mechanism to prevent uncontrolled inflammatory reactions. During activation of the inflammatory response, the influx of inflammatory cells is at its maximum, and the body can secrete a series of pro-resolving mediators (e.g., endogenous lipid-derived mediators) to clear inflammatory cells at the inflammatory center and inhibit PMN infiltration to prevent further damage [18, 25]. As a result, the inflammatory response can be controlled, homeostasis can be regulated, and an excessive inflammatory response and chronic inflammation can be prevented.

The resolution of inflammation is a sophisticated and active process. Its basic processes include: (i) limiting and inhibiting neutrophilic infiltration; (ii) modulating the secretion of chemokines and cytokines; (iii) inducing the apoptosis of neutrophils, and subsequently enhancing the endocytosis and efferocytosis of macrophages [26].

**Fig. 1:**
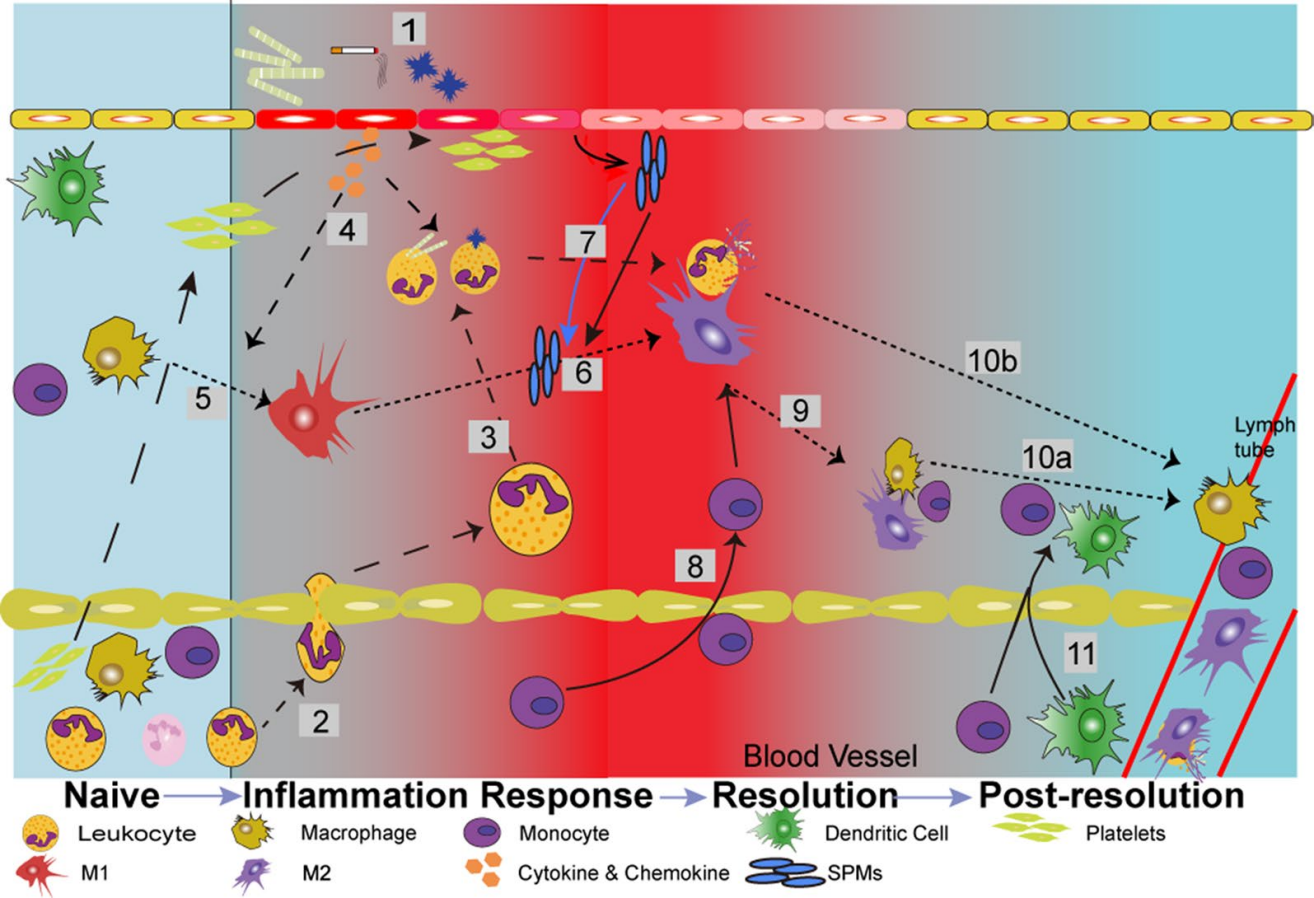
Processes of the inflammatory response and resolution. Extra stimulus will cause the injury of resident cells, such as epithelial cells, and cause the inflammation onset (1). These cells will be activated and release soluble pro-inflammatory mediators, which can mediate microvascular change, and promote leukocytes influx (neutrophils or eosinophils) (2). The basic function of leukocytes is to phagocyte and thereby to eliminate microorganisms and tissue debris (3). The resident cells and leukocyte in the inflammation center will produce cytokines to induce macrophage polarization and neutrophils recruit to the inflammation center (4,5). M1 cells will transform to M2 with the pro-resolution mediator to attend to the resolution phase (6). Neutrophil apoptosis (7) followed by efferocytosis (6,8) clear dysfunctional cells from the tissue. After clear the phagocytes, pro-resolving will promote macrophage program apoptosis. Excessive macrophage or non-apoptotic macrophage will leave the inflammatory site (9) or/and migrate to the lymphatic (10a,10b). At the end of inflammation resolution, resident immune cells regaining to form “adaptive immune” (11), which creates a status of “post-resolution”

However, regardless of how inflammation is activated and regulated, immune cells will be cleared from tissues. Briefly, during the inflammatory response, many PMNs, eosinophils, or lymphocytes are recruited to the inflammatory center under the induction of chemokines and cytokines, where they are involved in phagocytosis [26]. Thus, these cells become involved in local apoptosis or necrosis. Then monocyte-derived macrophages (MDMs) are also recruited as phagocytes to eliminate these apoptotic and necrotic cells [7]. Macrophages polarization and resolution cascade via the residence cells further produce pro-resolving molecules. Upon completion of exocytosis, macrophages can be removed from the inflammatory site through lymphatic drainage, thereby achieving the subsidence of inflammation. During regression, various complex signaling mechanisms and factors are required to control the balance between processes, including binding of cells to cell receptors and the secretion of endogenous lipid-derived mediators. If the signal-transduction mechanism is abnormal, the system of inflammation regulation will become disrupted, and inflammation will persist. This action will transform acute inflammation into chronic inflammation, which can lead to chronic damage, remodeling, and even fibrosis, and some tissues may lose their functions [27] (Fig. 1).

### Post-resolution of inflammation

Previously, the inflammatory response was thought to comprise two phases: inflammation and resolution. However, recent research [7, 28] has revealed that a third phase, known as “post-resolution”, can follow inflammation resolution, and thereby affect adaptive immunity and tissue healing [7]. Macrophages and DCs regulate this phase. Experiments have demonstrated that if mice are subjected to T-helper type 2 (Th2) inflammatory stimulation, then after inflammation has regressed, DCs in mice will not rapidly return to a stable state. [29]. In contrast, DCs show high expression of co-stimulatory signals, and stimulate mature DCs living in the airways for > 1 month [30]. Studies [31] have also shown that, in inflammation resolution, MDMs continue to exist stably in tissues for months. These adaptive immune responses function as “adapted homeostasis”, which can regulate the magnitude of the subsequent immune response. Therefore, this phase is considered to be an anti-inflammatory response to maintain immune cells, and may play a crucial part in determining the duration of acute innate immunity and the intensity of inflammatory stimulation.

## Regulation of lipid mediators in inflammation resolution

Lipid regulatory substances have essential roles as signaling molecules in inflammation because of their small size and specific effects on limiting infection. The most important lipid-derived mediators are SPMs, which include resolvins, LXs, protectins, maresins, annexins and some peptides [9, 27]. These substances are generated enzymatically *via* cyclooxygenase and lipoxygenase (LOX) pathways. They possess unique stereotypical characteristics, limit infection, and alter the lifespan of neutrophils by inhibiting their activation, migration, and infiltration. Also, SPMs and their regulatory cytokines can regulate adaptive immune system, promote the regression of inflammation, and re-establish tissue homeostasis [32, 33] (Table 1).

**Table 1:** Members of SPMs family and their roles in inflammation resolution

Mediator		Receptors	Functions	Refs.
*Alpha-linolenic acid (omega-3)*				
	Lipoxin A4	FPR2/ALX	Regulate leukocytes, PMNs, eosinophils, and monocytes	[177]
			Promote but inhibit apoptosis process	[178]
			Increase IL-10 production, and inhibit IL-6, IL-8, IL-12 expression	[39, 179]
			Inhibit the activation of the NF-kB pathway	[179]
			Regulate tight-junction formation in airway epithelial cells	[180]
	Lipoxin B4	GPCRs	Accelerate the regulation of allergic lung inflammation and airway hyper-responsiveness	[181]
			Inhibit the recruitment and transmigration of PMNs	[48, 182]
	Aspirin-triggered LX (ATL)	ALX/FPR	Inhibit neutrophil chemotaxis across the endothelium and epithelium	[37, 183]
			Enhance clearance and accelerate resolution of pulmonary edema	[2, 184]
			Reduce Cox2 traffic in pain responses	[2]
*Alpha-linolenic acid (omega-3)*				
EPA	RvE1	ERV1/ChemR23; BLT4 [185]	Mediate cell-migration and activation of the monocyte–macrophage system	[81, 185]
			Limit neutrophil infiltration, and promote macrophage phagocytosis	[186]
			Enhance phagocytosis and production of anti-inflammatory cytokines	[60, 61]
	RvE2	BLT1	Increase macrophage phagocytosis and efferocytosis	[45, 187]
			Regulate morphological changes as well as the chemotaxis and migration of PMNs	[45]
DHA	RvD1	DRV1/GPR32	Prevent T-cell differentiation towards Th1 and Th17 lineages	[188]
			Promote inflammation and enhance macrophage clearance	[45, 189]
	RvD2	DRV2/GPR18	Modulate expression of neutrophils, lymphocytes, and monocytes–macrophages	[48]
			Stimulate phagocytosis and apoptosis	[190]
	PD1	GPCRs	Inhibit neutrophil aggregation	[50, 51]
			Regulate the secretion of chemokines and cytokines	[50, 51]
			Transformation of mononuclear cells, macrophages, neutrophils and platelets	[182]
	Maresin	GPCRs	Inhibiting neutrophil migration	[13, 52, 53]
			Enhance recruitment of monocytes and macrophages and efferocytosis	[13]
			Inhibit IL-13 production and induce formation of regulatory T cells	[46]
			Induce polarization of M1 macrophages to M2 macrophages	[52]

### Lipoxins

LXs were first isolated and described in all SPMs by Chiang and Serhan in 1984 [34]. LXs, as a metabolite of the arachidonic-acid pathway, epithelial cells, neutrophils, leukocytes, and platelets are involved in its biosynthesis process. LXs can be synthesized by three major routes from arachidonic acid through three major lipoxygenases: 5-LOX, 12-LOX, and 15-LOX [9, 35, 36]. Leukotriene (LT) A4, LXA4, LXB4, and aspirin-triggered LX are synthesized starting from omega-6 arachidonic acid [37]. Considering LXA4 as an example [38], during the initial stages of inflammation, cells release the precursor protein AnxA1 of LXA4, which is an agonist that recognizes its specific receptor FPR2/ALX. The latter triggers the calcium-ion channels in cells to accelerate influx of calcium ions (Ca^2+^). The phospholipase A2, phosphoinositide 3-kinase (PI3K), and mitogen-activated protein kinase (MAPK) pathways become activated, promoting cell-surface phosphorylation FPR2/ALX receptor homodimer formation and pairs with LXA4. FPR2/ALX receptor on both phagocytes and monocytes phosphorated can induce cells to produce large amounts of interleukin (IL)-10 and promote the regression of inflammation [39]. LXA4 can inhibit the activation of serum amyloid A on the cell surface to delay apoptosis signals through phosphorylation via the FPR2/ALX pathway, and reduce the adhesion and aggregation of neutrophils [37, 40]. Otherwise, signals released by LXA4 can be combined with those of resolvin E1 to prevent an overflow of neutrophils, and thereby promote inflammation [41].

## Resolvins

Resolvins were first discovered in the exudates of mice during inflammation resolution [42]. They are derived from omega-3 polyunsaturated fatty acids in the form of eicosapentaenoic acid (EPA) and docosahexaenoic acid (DHA), which combine specifically with G protein-coupled receptors (GPCRs) and promote the elimination of bacteria and efferocytosis. Based on the different unsaturated fatty acids derived from it, EPA can be divided into resolvin E1 (RvE1) and RvE2, and DHA can be divided into resolvin D(RvD) and neuroprotectin [42]. Studies have shown that RvE1 mainly mediates the migration and activation of the monocyte–macrophage system by binding specifically with two types of receptors, ChemR23 and LTB4 receptor 1 (BLT1), to promote the resolution phase [43–45]. In addition, RvD1 helps to promote inflammation by interacting with FPR2/ALX and GPR32 receptors on the cell surface [46]. Its aspirin-triggered epimer, AT-RvD1, as well as RvD3 and RvD5, can bind the GPR32 receptor to express on human neutrophils, lymphocytes, monocyte–macrophages, and in vascular tissues [47]. RvD2 binds to DRV2/GPR18 receptors involved in the resolution of inflammation by modulating expression of neutrophils, lymphocytes, and MDMs [48].

### Protectins and maresins

Protectins and maresins are pro-inflammatory mediators derived from DHA [49]. Protectin is formed by the hydrolysis of DHA by LOX but the specific mechanism is incompletely understood. The process might be achieved through the inhibition of neutrophil aggregation and regulation of the secretion of chemokines and cytokines in combination with GPCRs to regulate the inflammatory response [50, 51]. Maresin (macrophage mediator in resolving inflammation, MaR) is a novel family of SPMs. This family mainly regulates the inflammatory response by limiting neutrophil migration and stimulates macrophage phagocytosis [52, 53]. Early production of MaR1 is dependent on platelet–neutrophil interactions to participate in inflammation regulation [54].

## Regulation of inflammatory cells

Neutrophils can survive only for several hours in peripheral blood. However, their survival can be extended by inflammatory mediators (IL-6, IL-8, or GMS-F) or lipopolysaccharide stimulation [7]. As inflammation progresses, neutrophils accumulate rapidly within the inflamed area and can digest the invading microbes. Then, the body restricts the influx of neutrophils to prevent persistent inflammation. This process, whereby the concentration gradient of chemokines and cytokines is regulated, activates the release of the pro-resolution mediators; thus, the recruitment and accumulation of neutrophils is no longer activated.

Neutrophils are the first line of defense against invading microbes. They are recruited rapidly to the center of inflammation to engulf microbial pathogens. While neutrophils engulf microbes and undergo apoptosis to break down into several fragments, they also become engulfed by macrophages, which promote resolution [55]. This complex regulatory process is carried out by signaling pathways, such as caspase-mediated endogenous or exogenous apoptosis mechanisms, and include other major pathways, such as PI3K/Akt, MAPKs, and B-cell lymphoma (Bcl)-2 [37]. The PI3K/Akt pathway can inhibit neutrophil apoptosis and promote inflammation by inhibiting the phosphorylation of apoptotic proteins. RvE1 and Mar1 can promote neutrophil apoptosis and inflammation regression by blocking the PI3K/Akt pathway.

In the MAPK pathway, extracellular signal-regulated kinase (ERK) and p38MAPK are considered important regulators of neutrophil survival [56]. Furthermore, RvE, Mar, and other SPMs can promote neutrophil apoptosis and inflammation resolution by inhibiting the phosphorylation of ERK and p38MAPK [57].

Research has demonstrated that apoptotic neutrophils can up-regulate the expression of many chemokines and activate monocyte–macrophage migration to apoptosis sites [7]. LXs and lactoferrin released by neutrophils can promote monocyte/macrophage migration and inhibit neutrophil migration [58]. These apoptotic neutrophils express various types of molecules on their surface, such as phospholipids and ribosomal proteins, which macrophages can recognize. Such molecules activate the phagocytic ability of macrophages, promote the rapid and effective clearance of apoptotic cells from tissues, prevent the initiation of a secondary inflammatory response and necrosis triggered by the release of toxic substances in neutrophils and, thus, promote the resolution of inflammation [59]. The phagocytosis and apoptosis of macrophages can increase the release of the pro-inflammatory lipid mediators LXA4, RvE1, PD1, and maresin. In addition, RvE1 can reduce the production of TNF-α and IL-1β, and increase the production of the anti-inflammatory factor IL-10 [60, 61]. Furthermore, RvD1 can increase the production of TNF-α and enhance the cell-killing ability of macrophages [62].

Neutrophils initiate antimicrobial and phagocytic activity against infection. Also, they release extracellular DNA complexes, which are called “neutrophil extracellular traps” (NETs), to regulate several infections [63]. In recent decades, NETs have been determined to play an important part in host defense and pathologic inflammation. Evidence shows that NETs are associated with pathophysiological processes in cardiovascular disease [64, 65], pancreatitis [66], asthma [67, 68], COPD [69, 70], and cancer [71]. As a protein–DNA complex, the main structures of NETs are extracellular DNA, histone, and many types of peptides, such as neutrophil elastase, myeloperoxidase, lactoferrin, gelatinase, and cathelicidins [72]. However, there still has little study about the action of SPMs on NET formation. A recent study demonstrated that LXA4 could decrease NETs release in vitro [73]. But NETosis will excessively produce after the lungs' bacterial infection in the ALX/FER2 receptor-deficient mice [73]. Moreover, RVD1 treatment of mice showed significantly low levels of NETS in aortic tissue of abdominal main pulse model animals [74]. Likewise, RVD4 treatment could decrease NETs release [74]. These studies show that SPMs might play roles in the formation of NETs and regulation in inflammation resolution.

During acute and chronic lung inflammation, many immune cells and structural cells undergo apoptosis progressively. The process by which phagocytosis (including that undertaken by macrophages, immature DCs, and atypical phagocytic cells) removes these apoptotic cells effectively is called “efferocytosis” [75, 76]. During the inflammatory response, the elimination of apoptotic cells is beneficial because it promotes the regression of inflammation and restores homeostasis in the lungs (Table 2). Specific signaling molecules are present in living cells, such as phosphatidylserine and other molecules, which are referred to as “eat me” molecules. While cells engulf pathogens and to initiate apoptosis, these cells are exocytosed and show high expression on the cell surface. Receptors on phagocytes can recognize these signals to enhance tissue homeostasis [77]. However, this effect is bidirectional; thus, phagocytes can induce the necessary cytoskeletal rearrangements to ingest the apoptotic cells and also transmit an instructive signal [76, 77].

**Table 2:** Different cell-type in inflammation resolution action

Cell type	General function	Actions in inflammation resolution	Ref.
Neutrophils	Phagocytosis Pro-inflammation NETosis	Accelerate cytokines and chemokines secretion Apoptosis NETosis Egress to Lymph node	[7, 51, 55]
Macrophages	PRR Phagocytosis Efferocytosis	M2 formation Effercytosis Secrete pro-inflammation cytokines, such as IL-10, TGF-β Egress to Lymph nodes Promote SPMs, including resolvins, maresins, protectins formation	[80, 81, 191]
Eosinophils	Phagocytosis Cytotoxic substances	IL-4, IL-13 secretion Lipoxin A4 production	[46]
Mast cells	Secretion of vasoactive substances	Mediators secretion	[51]
DCs	Sensing DAMPs PRR	TGF-b, IL-10 secretion Inhibits migration Maintain the homeostasis after inflammation resolute	[20, 21]
ILC2	Produce type 2 cytokines Express surface markers and receptors for chemokine	Inhibits IL-13 secretion, Express the chemokine receptors CXCR6 and CCR9 IL-25, IL-33, and thymic stromal lymphopoietin (TSLP) to induce inflammation formation and eosinophilic infiltration	[115, 117, 192]
Epithelial cells	Physical barrier Mucociliary clearance	Maintain mucosal integrity and to modulate local immune responses Decrease and limit pro-inflammatory mediators and proteins Increases proliferation after acid injury and promote tissue repair Immune regulator	[24, 193]
Endothelial cells	Regulation transduction and exudation	Inhibits TNF-a, IL-1b and IL-18 secretion Blocks the generation of reactive oxygen species	[51]
Fibroblasts	Tissue support Cytokine secretion	Growth factors inducement Inhibits CTGF-induced proliferation	[46, 194]

Macrophages can differentiate into M1 and M2 lineages under the stimulation of different microenvironments. M1 macrophages can express most of the toll-like receptors (TLRs) and secrete IL-12, TNF-α, IL-1β, IL-6, and other pro-inflammatory factors [78, 79]. During the early stages of inflammation, macrophage activation mediated by TLRs on RvD1 can increase the phagocytic ability of granulocytes and reduce the production of reactive oxygen species (ROS) to control the acute inflammation caused by oxidative stress and enhance the phagocytic function of macrophages [80]. Furthermore, RvE1 can stimulate the transformation of macrophages from the M1 phenotype to an intermediate phenotype. This is different from M2 polarization, but leads to secretion of anti-inflammatory factors such as IL-4, IL-10, and transforming growth factor (TGF)-β, and promotes inflammation resolution, vascular regeneration, and tissue repair [81].

If inflammation occurs in lung diseases, peroxisome proliferator-activated receptor (PPAR)-γ stimulates the transformation of macrophages from the M1 lineage to the M2 lineage through a complex regulatory mechanism [82]. Moreover, RvE1 can enhance macrophage efferocytosis on apoptotic neutrophils and bacterial fragments, induce the differentiation of non-inflammatory macrophages, and release several anti-inflammatory factors, such as TGF-β and IL-10, among others [60]. Thus, the environment in vivo can promote the conversion of the inflammatory response to inflammatory resolution and enhance host defense. In addition, efferocytosis can also increase the expression of prostaglandin (PG)E2 and some intracellular factors, which help to promote resolution and the restoration of homeostasis [83, 84].

## Resolution in diseases of the respiratory system

**Fig. 2:**
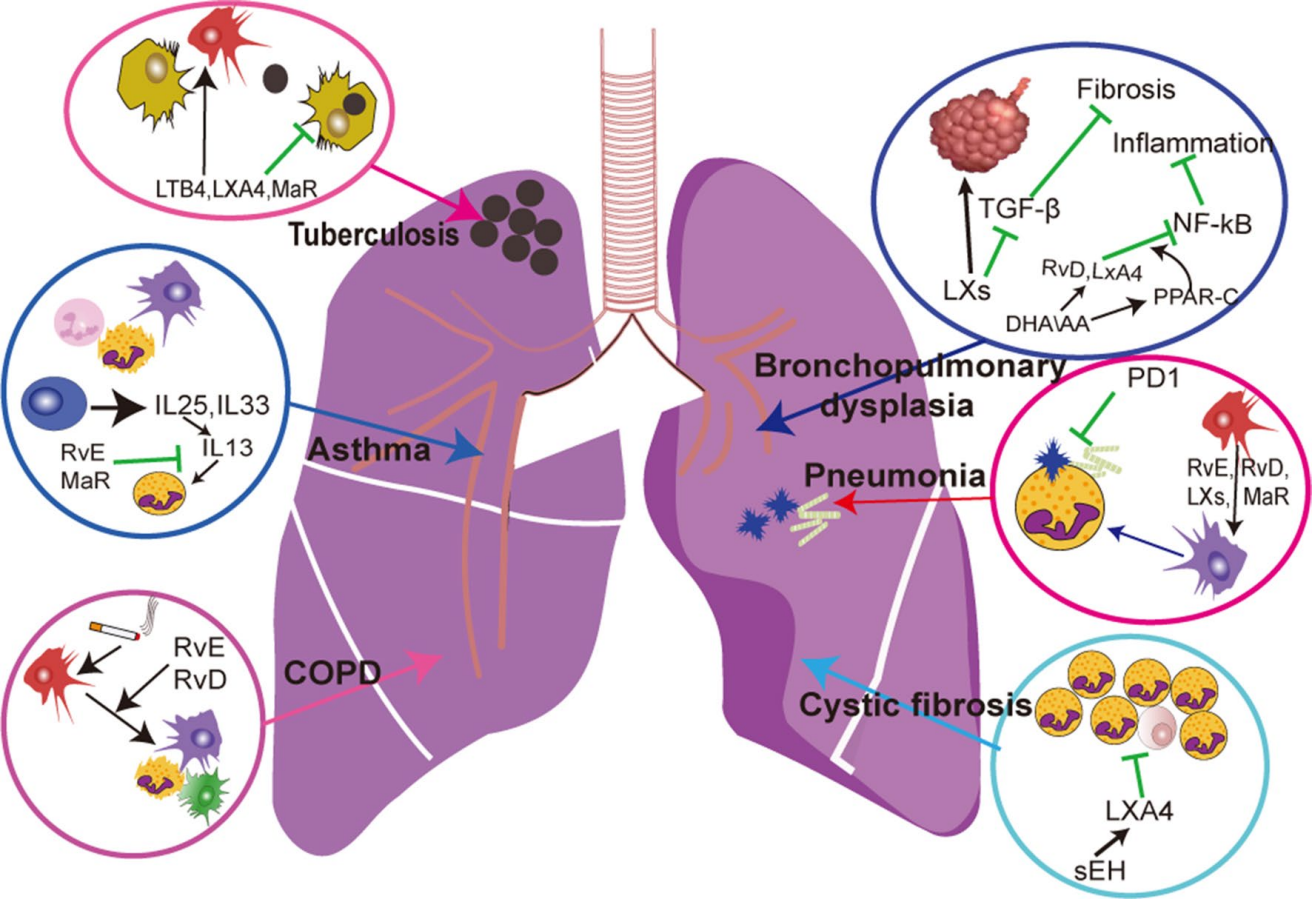
Cellular mechanism for SPMs in pulmonary disease. Specialized pro-resolving mediators (SPMs) contributed to the resolution of inflammation. These endogenous mediators involved in cytokines production, pathways regulation and inflammatory cell modulation, which can affect different pathophysiologic process of these pulmonary diseases. There is the major regulation pathway of SPMs in tuberculosis, asthma, COPD, cystic fibrosis and pneumonia and bronchopulmonary dysplasia

Pneumonia, acute lung injury (ALI), acute respiratory distress syndrome (ARDS), asthma, pulmonary fibrosis, and COPD are common respiratory system diseases and carry a high incidence and poor curative effect. These diseases can severely affect the quality of life, and may even lead to death. The mechanism of action of these diseases is, in general, associated with inflammation but the exact mechanisms have yet to be elucidated (Fig. 2). However, chronic, persistent, and unresolved inflammation is a key process in the formation of such chronic airway and lung diseases. In recent years, the relationship between these chronic airway diseases and chronic inflammation has been widely recognized. The study of respiratory diseases in inflammation subsidence has made initial strides.

### Pneumonia

Pneumonia is inflammation of the terminal airways, alveoli, and interstitium of the lungs. It can be caused by pathogenic microorganisms, physical and chemical factors, immune disorders, allergies, and drugs. Although most pneumonia is self-limiting, it can also be observed in chronic inflammatory foci. Furthermore, many pathogens, toxicants and allergens causing pneumonia or sepsis, which might induce acute respiratory distress syndrome (ARDS), and acute lung injury or inflammation (ALI). And the research demonstrates that these pneumonia- or sepsis-induced ALI/ARDS will promote different immune response and therapy effective [85].

A multicenter pilot study conducted in 2017 [86] reported that during community-acquired pneumonia, the levels of monounsaturated fatty acids (MUFAs) and polyunsaturated fatty acids (PUFAs) were increased significantly. The inflammatory response in the high-PUFA group of that study was increased even with a relatively moderate degree of inflammation. That finding suggests that unsaturated fatty acids have important roles during pneumonia progression. However, owing to the excessive lipid metabolism involved in the inflammatory response, PUFAs might not simply serve as an anti-inflammatory lipid medium in lung diseases.

Based on different stages of airway inflammation and disease severity, the pro-inflammatory resolution effects of PUFAs might be impacted by several factors [87, 88]. Epidemiological studies have revealed *Streptococcus pneumonia* to be one of the most common pathogenic bacteria in community-acquired pneumonia. A baboon model of pneumonia induced by *S. pneumoniae* infection found that the levels of endogenous lipid mediators (especially RvE1) in plasma were reduced during infection. The administration of exogenous RvE, such as lipoxins, considerably relieves among such mediators and promotes the repair of damaged lung tissue [89].

*Pseudomonas aeruginosa* is a common infectious factor in the respiratory system [90]. The complex antibiotic resistance make these bacterial results the acute exacerbation of COPD and mortality of Cystic fibrosis (CF). Animal experiments have shown that RvD1 expression is reduced significantly in pneumonia caused by *P. aeruginosa* infection [91]. Furthermore, RvD1 can significantly inhibit the activity of the TLR- MyD88/TRIF pathway by regulating the expression of TLRs and down-regulating the expression of microRNA (miR)-21 and 155 genes. In this way, RvD1 reduces the secretion of vascular endothelial growth factor (VEGF) and C-X-C motif ligand (CXCL), and thereby prevents excessive activation of macrophages and inhibits the expression of inflammatory factors [92]. Therefore, RvD1 can be regarded as a novel treatment candidate for persistent and refractory inflammation in the lung caused by *P. aeruginosa* infection owing to its cellular functions and molecular effects in host defense against pathogens in airway mucosal tissues.

In addition, LXA4 has protective effects on infected seed coats and endothelial cells, and promotes neutrophil apoptosis through the Bcl-2 pathway to reduce inflammation persistence [93]. Higgins and colleagues [94] demonstrated that LXA4 incubated with epithelial cells could inhibit *P. aeruginosa* infection-mediated expression of the tight-junction protein zonula occludens-1 on epithelial surfaces, which can protect and delay the bacterial’s invasion. This regulation could also enhance the barrier function by promoting tight junction formation and accelerating the epithelial repair.

In a mouse model of pneumonia induced by *Haemophilus influenzae* infection, Croasdell and coworkers [17] found that the inflammation and damage of pulmonary tissue were persistent, and was not alleviated even after bacteria had been cleared. However, the exogenous administration of 17R-RvD1 could regulate the transport of leukocytes to the lung. The resulting effects included reduction in the recruitment of neutrophils, acceleration of the influx of macrophages, the shifting of macrophage polarization, and reduction of the expression of IL-6 and TNF-α. These effects could reverse regulation of the inflammatory environment triggered by *H. Influenzae* infection [17].

Influenza viruses are the main causes of airway infection that can lead to severe pneumonia and, ultimately, death. Recently, Cilloniz and colleagues [95] found that enhancement of the pathogenicity of influenza viruses was positively correlated with the intensity of its inhibition of pro-inflammatory factors. The mortality rate could be reduced by injecting protective factors into mice infected with the H5N1 virus. This mechanism may be achieved by inhibiting RNA replication of the H5N1 virus [96]. Furthermore, PD1 has been demonstrated to be a potential drug to prevent the spread of the H5N1 virus. In a study of the H1N1 virus, Ramon and colleagues [97] found that the RvD precursor, 17(S)-hydroxydocosahexaenoic acid (17-HDHA), could promote a significant increase in the levels of anti-H1N1 antibodies in the serum, as well as increase the number of antibody-secreting cells in the bone marrow of mice.

Studies have demonstrated that LXs and protectin inhibit viral replication, reduce the severity of inflammation, promote neutrophil apoptosis, regulate neutrophil chemotaxis, and can reduce mortality significantly [98]. Although reports on the efficacy of SPMs on coronavirus infection-induced pneumonia are lacking, SPMs could be a potential treatment for retarding the associated inflammatory response and improving the prognosis, but this hypothesis merits further study.

### Tuberculosis (TB)

TB is associated with one of the highest numbers of deaths by infectious disease worldwide. According to the World Health Organization, there are > 10 million new cases of TB each year [99]. These increased morbidity and mortality levels from TB result from a complex disease process triggered by the pathogen Mycobacterium tuberculosis, which modulates inflammation at different stages intracellularly [100].

Granuloma formation is strongly correlated with inflammation regulation [101]. Local imbalance of inflammation results in extensive necrosis of granulomas and release of their liquefied contents into the bronchi and, thus, has dual roles in protection and disease [102]. Some inflammatory factors regulate chemokines and cytokines (e.g., IL-22), which prevent pathogenic epithelial cell-destructive inflammation by inhibiting the release of matrix metalloproteases and PMN-recruiting chemokines and promoting aberrant proliferation and differentiation of epithelial cells[103, 104]. Neutrophilic proteases can cleave IL-22R1 on epithelial cells and impair IL-22-dependent antimicrobial protein production from epithelial cells [105].

Studies [106–108] have shown that SPMs such as LTB4, LXA4, PGE2, and PGF2α play important parts in the susceptibility and pathogenesis of TB. Research on lipid-derived mediators suggests that LXs and maresin families are the most abundant pro-resolving lipid mediators in individuals suffering from TB [109]. Such mediators modulate the balance between pro-inflammatory and pro-resolving reactions. Furthermore, LXA4 might accelerate the progress of active TB. In contrast, PGs (especially PGE_2_ and PGF) play an important part in immunosuppression during infections [109]. However, the specific roles and functions of these SPMs in TB are not clear.

During TB progression, macrophages have key roles in inflammation regulation. PPAR-γ provides a major pathway in inflammation resolution, promoting the survival and growth of *M. tuberculosis* in macrophages in in vitro models, and can regulate the expression, polarization, and anti-inflammation of alveolar macrophages [110]. The latter also have potential roles in the promotion of *M. tuberculosis* growth in the lung [110, 111]. Research on TB treatment has demonstrated that the administration of LXs and PGE_2_ is useful in restricting the development of inflammation during the early phases [112].

### Asthma

Asthma could affect as many as 40 million people worldwide by 2025 [113]. Asthma obstructs airway ventilation, which is caused by chronic inflammation and hyperresponsiveness of the airways. Blood vessel proliferation in the bronchi, infiltration of inflammatory cells, mucus hypersecretion, and airway remodeling are the primary asthma pathophysiology [114].

Asthma onset can be divided into two phases [115]. Early asthma attacks could be considered to be due to acute inflammation of the airways [115]. If many allergens enter the body, they promote extensive accumulation of IgE, which mediates the degranulation of mast cells and eosinophils, and activates DCs and type 2 innate lymphoid cells (ILC2) to penetrate the bronchial mucosa. The release of LTs, histamine, PGs, and cytokines trigger an acute inflammatory response of the cells in the airways, thereby causing airway damage.

During the later phases of asthma, chronic airway inflammatory lesions may appear [115]. Many apoptotic epithelial cells and neutrophils accumulate in the airways of asthma patients. Eosinophils and macrophages begin to engulf and clear apoptotic cells in large quantities to maintain homeostasis. However, the number of airway macrophages in patients with severe asthma is reduced significantly compared with healthy people. Thus, complete clearance of apoptotic cells in patients with asthma is difficult, which might be related to a reduction in the production of anti-inflammatory eicosanoic acid or regulators of soluble hydrolysis. Lower levels of LXA4 in the serum, bronchoalveolar lavage fluid (BALF), and induced sputum are positively correlated with asthma severity and are closely related to oxidative stimulation and changes in lung function [116].

Studies on ILC2 in asthma have shown that it can stimulate epithelial cells to secrete the inflammatory factors IL-25 and IL-33, and mast cells to release PGD2, which can stimulate IL-13 secretion to induce bronchial hyperresponsiveness [115, 117]. Furthermore, IL-C2 promotes the recruitment of eosinophils by producing IL-5 [115]. Several pro-inflammatory factors (including eicosanoids, cytokines, chemokines, and growth factors that contribute to asthma symptoms and bronchial hyperresponsiveness) are secreted by activated epithelial cells and smooth muscle cells and may persist during chronic inflammation. In vitro, SPMs can inhibit the migration of eosinophils and neutrophils. Simultaneously, SPMs in combination with the expression of the transient receptor potential pathway can activate lung sensory neurons, and thereby regulate IL-C2 activity, promote the proliferation of bronchial epithelial cells, and enhance the repair and recovery of tissue [50, 51].

In addition, SPMs can inhibit lung fibrosis by inhibiting the proliferation of lung fibroblasts [101]. Animal experiments have demonstrated that PD1 can effectively inhibit the proliferation of eosinophils and T lymphocytes in the airways [118]. Maresin 1 and PD1 can inhibit the production of inflammatory factors (e.g., IL-5 and IL-13) [115]. Furthermore, PD1, like RvE1, is produced by increased levels of the chemokine receptor CCRX5 on the surface of apoptotic PMNs. Expression of maresin 1 is regulated by the interaction between TGF-β and IL-C2, which promote the dissipation of lung inflammation [10, 119]. In addition, treatment with RvE1 in allergic inflammation increases the formation of lipoproteins, directly inhibits the production of IL-23 and IL-17 in the lung, and promotes the cytotoxicity of natural killer cells [120]. These effects support the role of RvE1 in promoting the resolution of allergic pulmonary inflammation through other inflammatory pathways.

There are some patients with a low type 2 cytokine expression, which is called non-Th2 inflammation in asthma. This type of asthmatics always has a mixed granulocytic and chronic inflammatory response in the airway. Inadequately treatment with corticosteroids caused a persistent symptom and a higher exacerbation frequency [121]. For these patients, SPMs might combine with the receptors on NK cells or regulate eosinophilia and neutrophils' anti-inflammation function to inhibit the exuberant airway inflammation.

In an animal model of allergic asthma [122] characterized by eosinophilia, the TLR-7 receptor was able to recognize single-stranded RNA from the rhinovirus and self RNA during injury and necrosis of tissue. During the inflammatory response, the administration of TLR7 and other activators up-regulated DHA-derived SPMs such as PD-1, 17-HDHA, and 14-HDHA [123]. Therefore, the main mechanism of TLR7-mediated relief of airway inflammation is converting endogenous DHA to its bioactive products. However, the specific mechanism is not yet clear, and further research is needed. Recent research [46, 124] has shown that a continuous inflammatory response enhances oxidative stress in patients with severe asthma. Furthermore, the release of a large amount of soluble epoxide hydrolase can lead to reduced synthesis of 14,15-epoxyeicosatrienoic acid (EET), and a considerably reduced ability to synthesize LXs in the airways, which can reduce the resolution of lung inflammation.

Animal experiments have shown that LXA4 and its stable analogs have important effects on the prevention of allergic pulmonary inflammation. Furthermore, LXA4 can promote the repair of airway endothelial cells in asthma, up-regulate the expression of the tight-junction proteins occludin- and chudin-1, maintain transepithelial resistance, and maintain homeostasis of airway endothelial cells [125]. Simultaneously, LXs and LTs have a competitive inhibitory effect. Thus, LXs can effectively inhibit tracheal contraction, reduce airway hyperresponsiveness and mucous epithelial metaplasia, and promote the resolution of pulmonary inflammation [46, 115].

Glucocorticoids are considered to be the main anti-inflammatory drugs in asthma treatment. Glucocorticoids can activate ALX/FPR2 receptors jointly to regulate the high expression of anti-inflammatory factors, such as LXs and annexin A1 (which activates the adaptive immune response), increase the phagocytic capacity of macrophages, and reduce airway hyperresponsiveness [126]. Low-dose aspirin can reduce the expression of pro-inflammatory mediators, and can promote the expression of endogenous SPMs (e.g., resolvin and LXs), and thereby promote the regression of inflammation [127]. Furthermore, SPMs offer the potential to control inflammation and augment host defense in the non-Th2 inflammation in severe asthma patients. Some analogs and agonists of SPM have entered phase-I or -II clinical trials, and there is evidence that these drugs can promote the resolution of inflammation in asthma [128].

### COPD

COPD is a major pulmonary disease with high morbidity and mortality worldwide. COPD pathogenesis is associated with chronic and persistent inflammation in the lung, which correlates with obstruction and limitation of the small airways. The primary disease and inflammatory response in COPD can cause tissue damage and irreversible organ injury in such cases [129]. Current understanding of COPD is deepening, and the disease can now be divided into several phenotypes based on symptoms or tests, with a yet unclear pathogenesis.

A review by Barnes [130] showed that the major mechanism of airway obstruction in COPD is the loss of elastic recoil, which is caused by proteolytic destruction of the lung parenchyma. Thus, reversal of this condition with medication is usually impossible. Otherwise, preventing the occurrence and persistence of inflammation and enzymatic disease processes could inhibit COPD progression and prevent fibrosis [130]. Therefore, research into the various ways by which the subsidence of inflammation or the elimination of persistent external stimuli may be promoted would be helpful for the treatment of patients with early COPD or acute exacerbations of COPD, and could facilitate the repair of airway injuries.

The levels of RvD1 and LXs in the sputum, serum, and exhaled breath of COPD patients are reduced significantly compared with those in healthy people [131, 132]. The levels of LXA4 [133] and LXB4 in the exhaled breath of patients with moderate-to-severe COPD is reduced significantly, and is accompanied by a significant increase in the levels of pro-inflammatory factors [132]. The exogenous administration of RvD1 can significantly reduce the number of neutrophils and cells induced by the stimulation of cigarette smoke, while also reducing inflammation, oxidative stress, and even cell death [132, 134]. Those studies suggest that the persistence of chronic inflammation may destroy the pro-resolution pathway in the lung tissue of patients with COPD. SPMs reduce the incidence of emphysema by controlling chronic inflammation.

The transcription and synthesis of lipids is dependent upon several transcription factors, such as PPARs [135, 136], liver X receptors [137], and sterol regulatory element-binding proteins [138, 139]. These transcription factors may have particular importance in the pathophysiology or treatment of COPD and other diseases. In patients with COPD, PPAR-γ expression is down-regulated in lung tissue, epithelial cells, and myeloid DCs [140, 141]. Furthermore, PPAR-γ can promote polarization of M2 macrophages, which enhances the clearance of apoptotic neutrophils, and improves anti-inflammatory efficiency and the capacity for tissue repair [140, 142, 143].

Increased fibrosis in small airways is an important mechanism of disease progression in patients with COPD, and is speculated to be caused by chronic inflammation. This hypothesis suggests that efficacious anti-inflammatory treatment could prevent fibrosis [144]. Chronic persistent inflammation stimulates alveolar epithelial cells continuously to cause repeated tissue repair and scar formation. Eventually, the movement of the alveolar wall becomes restricted, and the small airways become stenosed, which limits gas exchange and promotes emphysema.

In addition, the polarization of macrophages under the stimulation of cigarette smoke plays an important part in COPD pathogenesis. Under the stimulation of cigarette smoke, the classical pathway of macrophages is activated to produce M1 macrophages, which secrete pro-inflammatory factors. If M2 macrophages are activated through alternative pathways, they can effectively remove inflammatory cells and apoptotic cells from the airways, and promote the subsidence of inflammation as anti-inflammation factors [145, 146].

Studies have shown that resolvins can induce macrophages to differentiate into the M2 lineage [132]. Studies on cigarette smoke-stimulated macrophages have shown that RvE1 promotes macrophages recruitment, significantly up-regulates expression of anti-inflammatory factors, and reduces levels of inflammatory response factors in culture supernatants [147]. Similarly, RvD1-supplemented macrophages are significantly activated to the M2 type, which occurs concomitantly with an increase in IL-10 secretion and significant improvement in the phagocytic capacity of macrophages [148]. Therefore, SPMs may play an important part and have emerging therapeutic importance in controlling COPD onset caused by cigarette smoke via the induction of macrophage polarization.

### ALI/ARDS

A severe injury or infection in the lung can activate leukocyte recruitment, and preserve tissue homeostasis. However, an over-reactive inflammatory response might increase the exosmosis and infiltration of leukocytes in normal tissue. This observation suggests that an excessive inflammatory response can cause secondary lung injury and may even cause ARDS, a common (but acute) disease with a high mortality. Several methods have been used widely in ALI treatment but few are useful during early inflammation. In addition, sepsis and systemic inflammation are related to the pathogenesis of ALI/ARDS [149]. SPMs can potentially regulate the systemic inflammatory response and inhibit sepsis. They have been demonstrated to be the major factors in how the inflammation wrought by ALI/ARDS is resolved [54]. Therefore, SPMs can potentially promote regression as a new treatment regimen for ALI/ARDS.

During the early phases of ALI, disease progression is mediated by the interaction between platelets and neutrophils, which promote the secretion of LXs and maresin 1 [54, 150]. According to research conducted on BALF, exogenous AT-RvD1, AT-RvD3, and RvE1 help to prevent continuous damage to pulmonary tissue. This is achieved by reducing the number of inflammatory cells exuded (especially leukocytes), reducing the release of pro-inflammatory factors, and repairing bronchial epithelial cells [151, 152]. In addition, AT-RvD1 and RvE1 activate and polarize macrophages to the M2 lineage, thereby accelerating bacterial phagocytosis [153]. Qi and colleagues showed that LXA4, as a major regulator of the miR-21/PTEN-AKT pathway, can promote sodium ion (Na^+^) entry into type-II alveolar cells by up-regulating the expression of subunits δ and γ proteins on Na^+^ channels and aquaporin 5, thereby increasing clearance of alveolar fluid [154].

### Cystic fibrosis (CF)

A single gene mutation called the cystic fibrosis transmembrane conductance regulator (CFTR) causes CF formation. CF leads to increased secretion of respiratory mucus, recurrent airway infections and, eventually, pulmonary failure. Genetic studies of CF patients have shown that they carry a gene polymorphism encoding prostaglandin-endosperm synthase 2, which reduces the secretion of pro-inflammatory factors and improves the clinical condition [155]. LXs can inhibit neutrophil infiltration, remove pathogenic bacteria, and effectively improve CF in mice. Like asthma and COPD, the expression of LXA4 in the BALF of patients with CF is also reduced significantly [98, 156]. Furthermore, levels of DHA and its derivative, Mar1, are also reduced significantly in patients with CF [157, 158].

Studies on lipid-mediated agents in the airways of patients with CF have shown lower levels of lipoproteins and SPMs compared with those in healthy controls [157, 158]. In addition, patients with detectable levels of RvE1 in the respiratory tract have better lung function than patients without detectable levels of RvE1 [156]. Moreover, in an animal model of CF, the exogenous administration of soluble epoxide hydrolase has been shown to inhibit the intracellular levels of 15-epi-LXA4, reduce neutrophil infiltration into the airways, and improve lung function [159, 160].

### Bronchopulmonary dysplasia (BPD)

BPD is common in premature infants. The mortality associated with BPD is high, and even survivors might live with various sequelae that could affect basic language skills or cognitive function. BPD pathogenesis is not clear but early supplementation with PUFAs may play a role in promoting lung maturity in infants [161, 162]. DHA and arachidonic acid can stimulate PPAR-C and/or directly synthesize RvD1 and LXA4 by inhibiting activation of nuclear factor-kappa B [163, 164]. These actions stop the inflammatory response, reduce the lung injury caused by hyperoxia, and delay BPD development [163, 164]. Although RvD1 can promote the dissipation of lung inflammation, it cannot affect the production of other VEGFs or members of the TGF-β family, nor the expression of BMP/Smad signaling proteins under high levels of oxygen exposure [165, 166].

Furthermore, LXA4 can promote the formation, reduce the average spacing between, and effectively increase the number of alveoli [167, 168]. In addition, TGF-β expression in newborn mice exposed to hyperoxia is decreased in the initial stages BPD, and is increased subsequently as the disease progresses. The LXs that regulate the TGF-β signaling pathway not only inhibit the proliferation of NIH/3T3 fibroblasts [169], but also down-regulate expression of the factors related to pulmonary fibrosis (e.g., collagen I, elastin, lysine oxidase-2, matrix metalloproteinases-2 and 9, and tissue metalloproteinase inhibitor-11) to exert protective effects in BPD induced by hyperoxia in newborn mice [170–172].

## Conclusions

Inflammation response and resolution are complex processes which involve a huge number of immune cells and regulatory factors. The dysregulation of inflammation resolution is the primary pathophysiology of disease development. Therefore, the direct or indirect clearance of pathogens via all types of immune cells limits any overlap between inflammatory phases and prevents chronic inflammation development. SPMs family, their pathways, and receptors could provide a basis for new approaches for treating inflammation-associated diseases, especially in respiratory diseases.

Pneumonia and TB are caused by various microbial factors and produce a complex immune response. Many factors can cause pneumonia. Over the past few decades, coronaviruses have repeatedly posed a public health concern. The coronavirus infection-induced alveolar, lung tissue, and extra-pulmonary damage are major caused by the “inflammatory storms” and immune responses. They also cause higher mortality rates than those elicited by pneumonia due to infection with influenza viruses. Continuing research on coronaviruses has shown that they can elicit an early, rapid immune response. The inflammatory storm might cause lung tissue damage, impair function, and reduce vital capacity. In addition to the pathogenicity of the virus, the body’s inflammatory response also has a vital role in cases of coronavirus infection-induced lung injury [173]. Recent studies show that Omega-3 fatty acids, specifically EPA and DHA, could reduce the risk of ARDS and the need of intensive care unit (ICU) admission in coronavirus disease 2019(COVID-19) [174, 175]. Moreover, a traditional Chinese medicine research has shown that the inhibition of the arachidonic-acid metabolic pathway potentially inhibits the release of inflammatory factors and alleviates the “cytokine storm” in the early infectious phase [176]. These researches suggest that SPMs might be a novel target for the rapidly inflammation reaction in the future.

Otherwise, SPMs such as LXA4, RvE1, and maresins are potential receptors to limit tissue injury in diseases of persistent inflammation. Allergies and chronic inflammation in patients with asthma or COPD should be treated with SPMs to modulate the activity of neutrophils, eosinophils, NETosis and macrophages, and thereby restore host homeostasis. Other lung diseases, including CF and BPD, show complex pathophysiology. Nevertheless, SPMs target receptors or regulate downstream processes, such as those associated with EET, to modulate inflammation pathways during the early phases of disease to prevent further progression.

In this review, we summarize the mechanism and research process of SPMs in inflammation resolution, especially in pulmonary disease. With the deep research on SPMs, the role of SPMs on inflammation resolution in different inflammation phases has been confirmed. Each SPMs potentially affects the inflammatory response and resolution, and even participation in tissue repair. Therefore, the anti-inflammatory potential of SPMs and their analogs could be a novel therapeutic approach for the treatment of chronic pulmonary and respiratory tract diseases in the future.

## Data Availability

Not applicable.
